# Beyond the Score: Fixed-Budget Benchmarking of Virtual Screening Integration Strategies for Decision-Centric Drug Discovery

**DOI:** 10.3390/ijms27167497

**Published:** 2026-08-21

**Authors:** Elisabetta Grazia Tomarchio, Rocco Buccheri, Antonio Rescifina

**Affiliations:** 1 Department of Drug and Health Sciences, University of Catania, Viale A. Doria 6, 95125 Catania, Italy; elisabetta.tomarchio@phd.unict.it (E.G.T.); rocco.buccheri@unict.it (R.B.); 2 Department of Biomedical and Biotechnological Sciences, University of Catania, Via Santa Sofia 97, 95123 Catania, Italy

**Keywords:** computer-aided drug discovery, machine learning, molecular docking, structural similarity, QSAR, virtual screening, fixed-budget benchmarking, hit recovery

## Abstract

Virtual screening (VS) workflows often combine structure- and ligand-based methods; however, their value depends on the number of compounds that can be tested. We benchmarked 20 fixed-budget strategies derived from molecular docking (GNINA CNN score), maximum common substructure (MCS) similarity, and a calibrated machine-learning (ML)-QSAR classifier across five pharmacologically diverse targets. Individual methods, best-rank and worst-rank fusion, mean-rank consensus, and sequential funnels were evaluated at 1%, 5%, and 10% library fractions, with every strategy selecting the same number of compounds. ML-QSAR was the strongest standalone method, recovering 47.6%, 81.6%, and 84.4% of actives at the three cutoffs. At the 1% budget, ML-QSAR achieved the highest mean hit recovery (47.6% recall; 99.2% precision). At 5% and 10%, best-rank fusion of QSAR and MCS produced the highest mean recall (83.2% and 86.4%). Among the sequential workflows, QSAR → MCS achieved the highest 1% hit recovery (45.2 ± 3.3% recall), whereas docking-first funnels consistently underperformed under the default, non-optimized conditions evaluated in this study. Target-level results showed substantial variability in MCS-containing workflows and limited benefits from adding docking without target-specific optimization. Under matched assay budgets, a validated ligand-based predictor or a simple two-method rank-fusion scheme provided the highest observed mean hit recovery without requiring elaborate integration.

## 1. Introduction

Over the past two decades, computer-aided drug discovery (CADD) has transformed early-stage drug discovery by enabling the rapid evaluation of millions of compounds at a speed and cost unattainable through experimental screening alone [1,2]. Virtual screening (VS) has become a central component of this process, integrating structure-based docking, ligand-based similarity approaches, and machine learning (ML) models to prioritize chemical space and identify promising candidates [3,4,5]. However, as these methods have matured, the main challenge has shifted from generating predictions to making reliable decisions based on them. In practice, experimental validation remains constrained by time, cost, and throughput, making the efficient prioritization of compounds a critical step in the discovery pipeline.

From a medicinal chemistry perspective, the objective is rarely to identify a single highest-scoring molecule. Instead, the goal is to select a small subset of compounds with the greatest likelihood of containing true actives within a limited experimental budget. In the present study, the term “experimental budget” refers specifically to the number of compounds that can be advanced to experimental testing, rather than to monetary or computational cost. Consequently, the practical value of a VS workflow depends less on global performance metrics, such as the area under the ROC curve (AUC) or affinity prediction accuracy, than on its ability to achieve high early enrichment while minimizing false positives among the top-ranked candidates [6,7,8]. This shifts the focus from score-based ranking to decision-oriented compound selection, where the outcome of interest is the quality of the compounds selected for experimental testing.

Despite the broad availability of open-source computational methods [9], their prospective use in medicinal chemistry remains limited. In many medicinal chemistry groups, particularly where computational expertise is limited, docking and QSAR models are still used primarily to rationalize experimental results rather than to support early compound prioritization and decision-making [10]. This limited adoption reflects several factors, including variable predictive performance across biological targets, concerns about the generalizability of ML models, and the lack of practical guidelines for integrating heterogeneous computational approaches into coherent screening workflows. Consequently, computational methods that can substantially reduce the chemical search space are often underutilized, even though recent advances in deep learning scoring functions and ensemble learning have significantly improved their predictive performance [11,12].

Most benchmarking studies have evaluated docking [13], ligand-based similarity [14], and QSAR models independently [15], providing valuable insights into the strengths and limitations of each approach. However, this does not fully reflect real-world drug discovery, where multiple computational methods are routinely combined to improve hit identification [16,17]. Moreover, many published benchmarks rely on proprietary software, limiting their reproducibility and applicability to new projects in the field.

To address these limitations, reproducible benchmarks based on open-source tools and standardized protocols are required. Evaluating integration strategies under default, non-optimized conditions provides a realistic assessment of the performance that can be achieved by research groups without access to extensive computational resources or target-specific optimization, thereby offering guidance that is directly transferable to routine medicinal chemistry applications.

**Figure 1:**
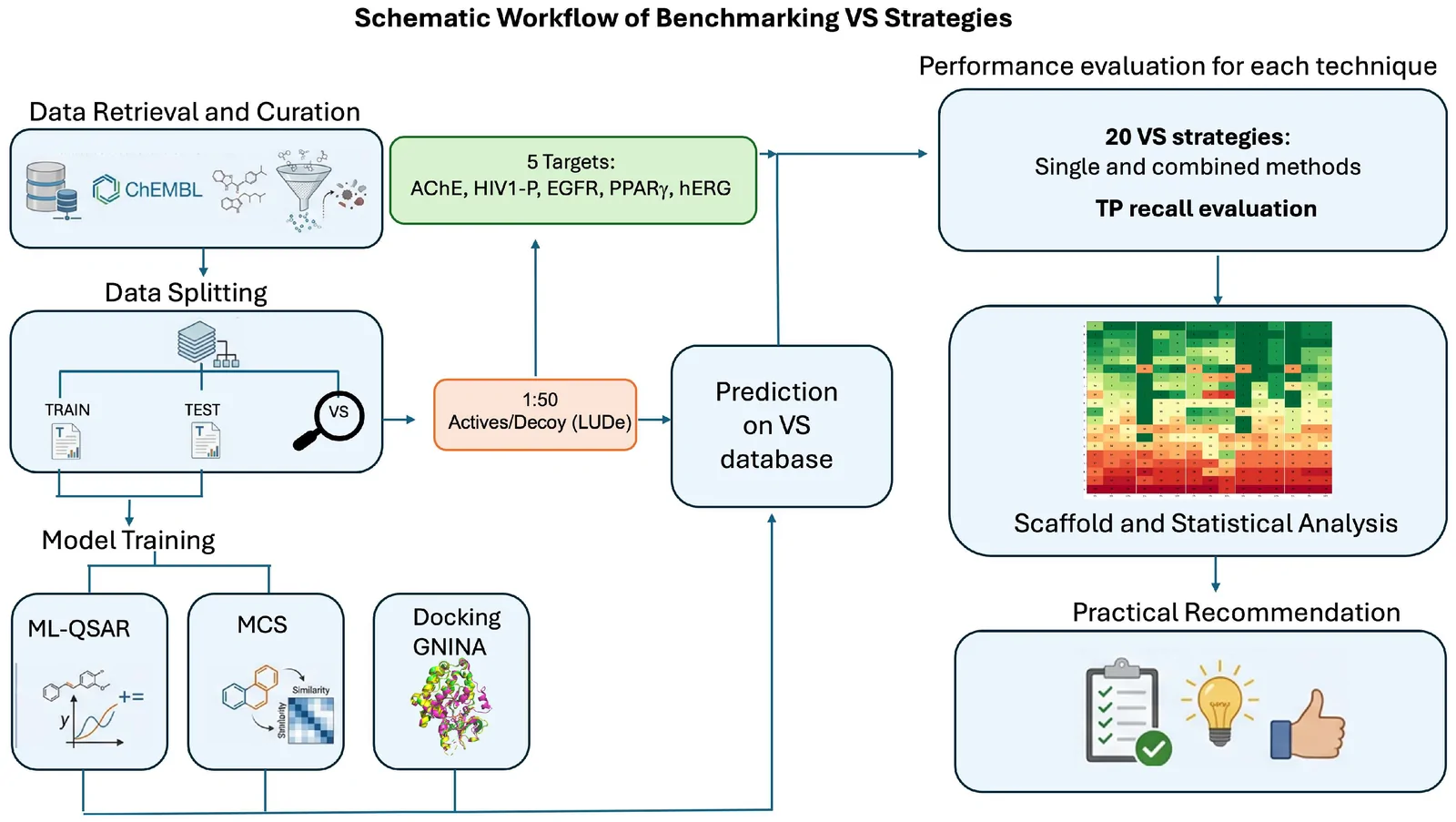
Schematic representation of the workflow employed in this study.

Here, we present a systematic benchmark of 20 VS strategies integrating three complementary reproducible approaches: structure-based docking using a convolutional neural network scoring function (CNN score), maximum common substructure (MCS) ligand similarity [18], and an ensemble ML-QSAR classifier. The strategies encompass four integration schemes: best-rank and worst-rank fusion, mean-rank consensus, and sequential funnels. They were evaluated across five pharmacologically diverse protein targets: acetylcholinesterase (AChE), epidermal growth factor receptor (EGFR), HIV-1 protease (HIV1-P), peroxisome proliferator-activated receptor gamma (PPARγ), and the human Ether-à-go-go-Related Gene (hERG) potassium channel. Most benchmarking studies compare integration strategies using identical score thresholds but allow each workflow to produce candidate lists of different sizes. Because experimental screening capacity is typically fixed, unequal list sizes confound methodological performance with an experimental budget. To address this limitation, all workflows in the present study were compared under an identical compound budget. A central feature of this framework is that all individual and integrated methods are evaluated head-to-head on the same compound libraries and under the same final selection size, so that differences in hit recovery reflect the prioritization strategy rather than differences in the number or identity of compounds available for evaluation. Performance was assessed at experimentally relevant screening fractions (1%, 5%, and 10%) using precision and recall as decision-oriented metrics. By focusing on compound selection rather than prediction alone, this study provides practical, evidence-based recommendations for designing effective and reproducible VS workflows suitable for early-stage drug discovery (Figure 1). To our knowledge, this study provides the first systematic benchmark comparing heterogeneous VS integration schemes, including rank-fusion and sequential integration strategies, with corresponding single methods under a matched experimental compound budget across multiple targets. Fixed-budget considerations are already implicit in early-recognition and enrichment-based evaluation, where performance is assessed at predefined list sizes, while hierarchical VS workflows commonly impose downstream selection limits at successive screening stages. However, these studies generally address evaluation metrics or individual screening architectures rather than systematically benchmarking alternative integration operators under the same final experimental budget across multiple targets.

## 2. Results and Discussion

The objective of this study was to determine which individual or integrated workflows recovered the largest number of actives at a fixed experimental budget and whether their relative performance remained consistent across targets and budget levels. Because the final selection size was fixed for each target and cutoff, both recall and precision were determined directly by the number of recovered actives; therefore, equivalent rankings of the strategies were provided. Accordingly, recall was selected as the primary outcome for comparative visualization and statistical analysis, while precision was retained for completeness in the accompanying tables. Single-method configurations were evaluated using their predefined scoring procedures rather than undergoing extensive target-specific hyperparameter optimization, thereby maintaining a consistent basis for comparison with the integration strategies. The results and their interpretation are presented together below.

### 2.1. Dataset Composition and Benchmark Design

The benchmark comprised five structurally and pharmacologically diverse targets: AChE, EGFR, HIV1-P, PPARγ, and hERG potassium channels. For each target, structure-based docking (CNN score), MCS similarity, and ML-QSAR prediction probabilities were compared. The VS datasets comprised 2345–2549 compounds per target (mean 2467), each containing 50 experimentally confirmed actives; the corresponding fixed budgets were 23–25, 117–127, and 234–254 compounds at the 1%, 5%, and 10% fractions, respectively. The training and test metrics for each method are provided in the Supplementary Materials. Twenty strategies were evaluated at three screening fractions (1%, 5%, and 10%), generating 300 target-specific measurements that were summarized as mean ± standard deviation (SD) across the five targets (Supplementary Materials). Because each VS library contained 50 confirmed actives while the 1% budget allowed only 23–25 compounds to be selected, the maximum recall attainable at this cutoff was 48.0% on average across the five targets. Recall values at the 1% fraction must therefore be interpreted against this ceiling rather than against 100%; at the 5% and 10% budgets, the budget exceeds the number of actives, and no such ceiling applies.

### 2.2. Individual Scoring Methods: QSAR as the Core Component

**Figure 2:**
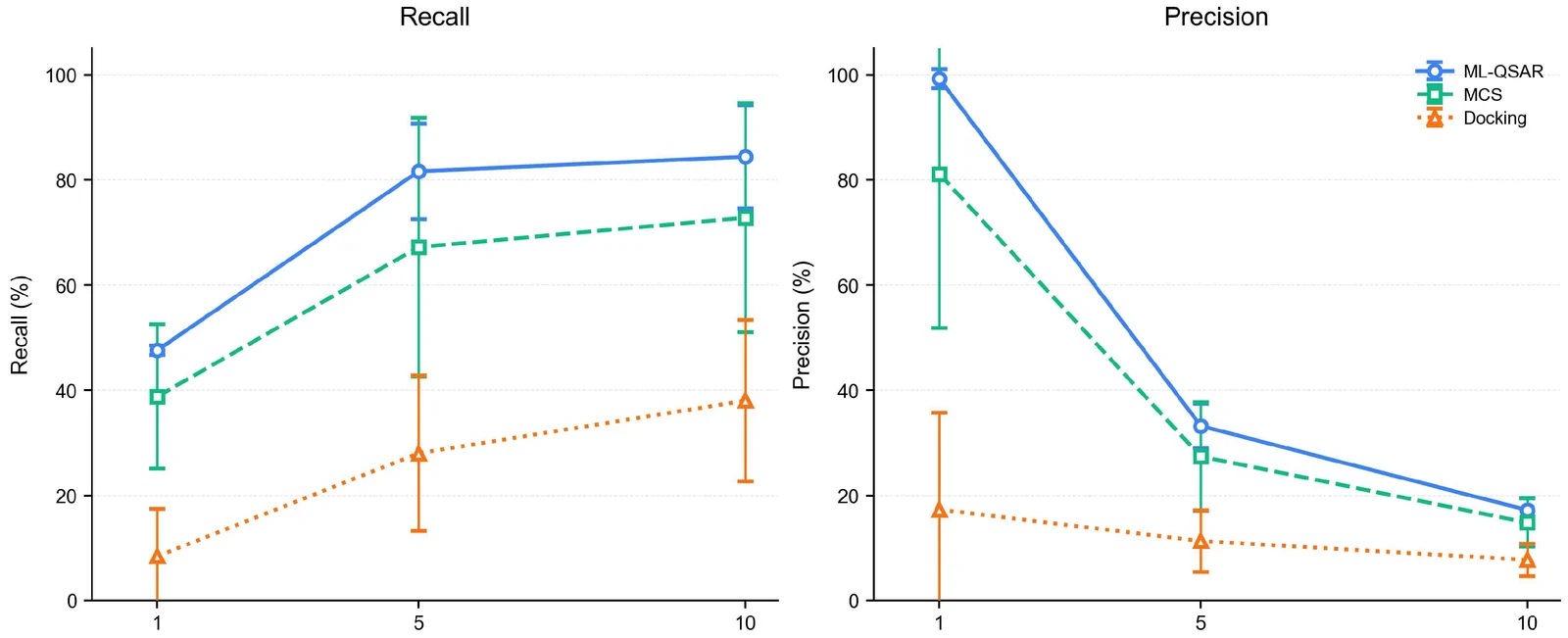
Performance comparison of the three VS strategies across screening cutoffs at a fixed budget. Recall and precision obtained by ML-QSAR, MCS similarity, and molecular docking at the top 1%, 5%, and 10% of the ranked libraries. All methods selected the same number of compounds, enabling a direct comparison of recall and precision. Values are mean ± SD across the VS dataset. Error bars represent standard deviation across the five benchmark targets and should not be interpreted as confidence intervals.

Among the individual approaches, the ML-QSAR classifier consistently achieved the best performance across all screening fractions, recovering 47.6 ± 0.9%, 81.6 ± 9.1%, and 84.4 ± 9.8% of confirmed actives at the 1%, 5%, and 10% cutoffs, respectively (Table 1, Figure 2). At the 1% cutoff, the precision remained exceptionally high (99.2 ± 1.8%), corresponding to 99.2% of the maximum recovery attainable within that budget, indicating almost no false positives among the top-ranked compounds. MCS similarity produced intermediate performance, with recall increasing from 38.8 ± 13.7% at 1% to 72.8 ± 21.8% at 10%; its larger variability across targets reflects the dependence of ligand-based similarity on the size and chemical diversity of the reference active set. Docking with the CNN scoring function showed the lowest recall, ranging from 8.4 ± 9.0% at 1% to 38.0 ± 15.3% at 10%, with substantial target-to-target variability.

These results indicate that fingerprint-based machine learning constitutes the best-performing standalone approach when a sufficient set of annotated compounds is available. Its high recall, near-perfect precision at the 1% cutoff, and low inter-target variability are consistent with previous reports on the strong performance of ensemble-learning models in ligand-based VS [19]. The comparatively low recall of docking should not be interpreted as a limitation of structure-based VS itself, but rather as a consequence of the deliberately reproducible, minimally optimized workflow adopted here, because docking performance depends strongly on protein structure quality, binding site definition, and protocol optimization. To verify whether the performance penalty observed for docking was strictly driven by the default protocol or robust to reasonable parameter variations, a sensitivity analysis was conducted on a representative target, AChE. Three docking conditions were evaluated under the same fixed experimental budgets (1%, 5%, and 10% fractions): Protocol A (the default workflow with a wide grid box and an exhaustiveness of 8), Protocol B (the default wide grid box with an increased exhaustiveness of 32), and Protocol C (a focused grid box tightly centered on the co-crystallized ligand with an exhaustiveness of 32). As detailed in Table S1, both increasing the sampling exhaustiveness (Protocol B) and focusing on the search space (Protocol C) provided modest improvements in true positive recovery and recall compared to the default baseline. Nevertheless, the absolute recovery rates remained substantially lower than those achieved by ligand-based approaches at matched budget fractions. These results show that reasonable increases in sampling and focusing of the binding-site search space partially improve standalone docking performance but do not eliminate its recovery gap relative to the ligand-based methods for the AChE test case. Because the integrated docking-first funnels were not re-evaluated under the optimized settings, the sensitivity analysis should not be interpreted as a direct test of their protocol robustness. Under these strictly standardized, non-optimized conditions, docking is more valuable as a complementary structural filter than as a primary ranking method, establishing ligand-based prediction, particularly ML-QSAR, as the natural backbone of the screening pipeline.

### 2.3. Fixed-Budget Performance of Integration Strategies

**Figure 3:**
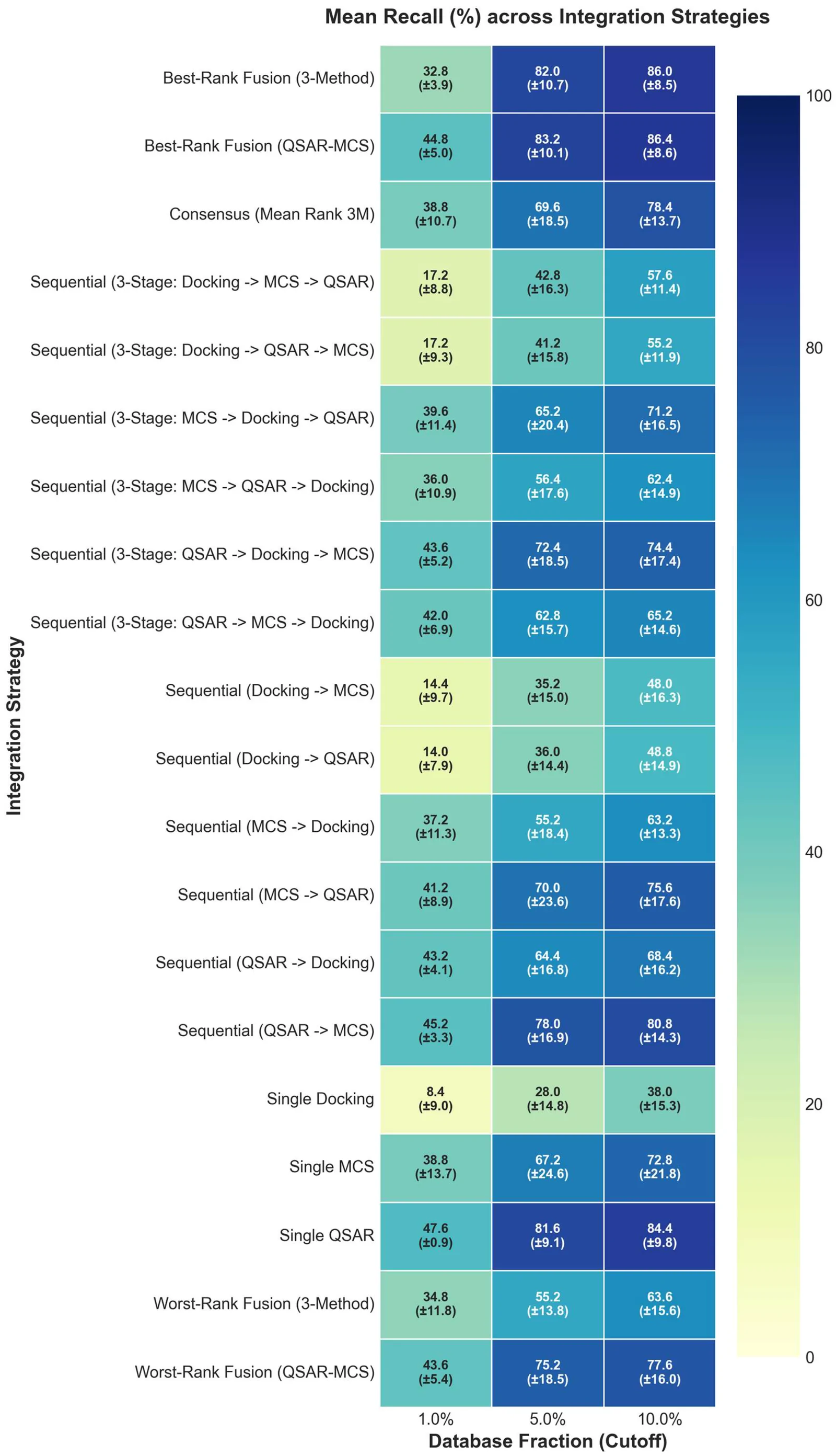
Comparison of VS integration strategies under a fixed experimental budget. Mean recall (%) at screening cutoffs of 1%, 5%, and 10% across all benchmark targets. Each integration strategy was constrained to select the same number of compounds at every database fraction. Cooler colors indicate higher performance.

Combining the three methods substantially reshaped the hit recall relative to the individual predictors. The multi-method workflows were grouped into four integration schemes: best- and worst-rank fusion, mean-rank consensus, and sequential funnel. (Figure 3).

To enable a fair comparison, all strategies were evaluated under a fixed experimental budget, with each workflow prioritizing the same number of compounds at each screening fraction (1%, 5%, and 10% cutoffs). Consequently, differences in recall and precision reflect the effectiveness of the integration strategy itself rather than differences in the number of selected candidates.

Among the best-rank fusion strategies, QSAR–MCS best-rank fusion achieved the highest recall at the 5% and 10% cutoffs (83.2% and 86.4%), narrowly exceeding the three-method best-rank fusion (82.0% and 86.0%); at the 1% cutoff, standalone QSAR alone achieved the highest recall of any strategy (47.6%), exceeding all union workflows. Adding docking to the best-rank fusion, under the evaluated protocol, did not improve recall at any cutoff, likely because the weaker predictor diluted the combined rank. Among the worst-rank fusion strategies, the QSAR–MCS combination recovered 75.2 ± 18.5% and 77.6 ± 16.0% of the actives at the 5% and 10% budgets, respectively; however, it remained inferior to QSAR–MCS best-rank fusion at both cutoffs. Among the sequential workflows, QSAR → MCS achieved the highest performance at the 1% budget, with 45.2 ± 3.3% recall and 94.3 ± 8.8% precision (Figure S1). Workflows initiated by docking consistently recovered fewer actives than the corresponding ligand-based funnels. This pattern was observed for both two-stage workflows (Docking → MCS and Docking → QSAR) and the two docking-first three-stage orderings. The mean-rank consensus produced intermediate performance, reaching 78.4 ± 13.7% recall and 16.0 ± 3.1% precision at the 10% cutoff.

### 2.4. Fixed-Budget Hit Recovery and Cross-Target Strategy Ranking

Because the selected set size |S| was fixed to the same budget B for every strategy within a given target and screening fraction, and A was likewise fixed per target, precision and recall were algebraically coupled (Precision = (A/B) × Recall): both metrics were monotonic transformations of *a_s_*, the number of recovered actives. Therefore, the matched-budget comparison is best interpreted as a ranking of true-positive recovery at equal assay capacity, rather than a conventional precision–recall trade-off.

At the 1% budget, the candidate list is smaller than the number of actives, so several strategies reached the same budget-limited maximum: eight to 12 strategies recovered an identical number of actives for HIV1-P, EGFR, and PPARγ. Therefore, the rankings at this cutoff are partly saturated, and the relevant observation is that ML-QSAR was the only strategy attaining the per-target maximum for all five targets.

**Figure 4:**
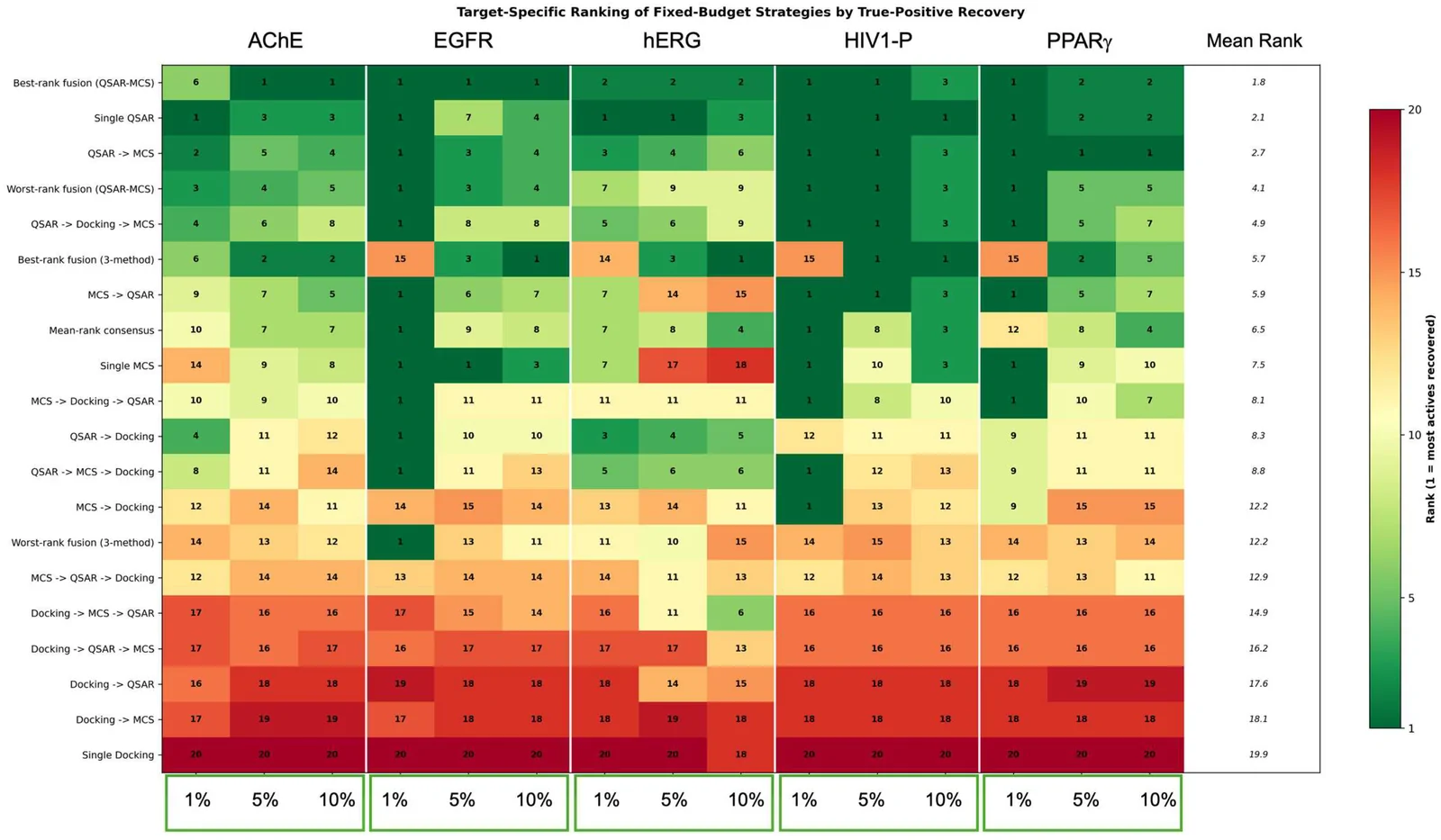
Target-specific ranking of the 20 fixed-budget strategies according to the number of true positives (TP) recovered. For each target and screening fraction, rank 1 denotes the largest TP count, with tied strategies receiving the same rank. The heatmap summarizes cross-target consistency and identifies workflows whose relative performance depends on the biological target or the assay budget.

At the 1% budget, single-method ML-QSAR achieved the highest across-target mean recall (47.6%) and was tied for the largest number of recovered actives for all five targets (Figure 4). At the 5% and 10% budgets, QSAR–MCS best-rank fusion yielded the highest across-target mean recall, reaching 83.2% and 86.4%, respectively, slightly ahead of the three-method best-rank fusion, which achieved a somewhat lower mean recall but remained competitive for several individual targets, indicating target-dependent complementarity that should be confirmed in a broader benchmark dataset. Sequential funnels initiated by docking (Docking → MCS, Docking → QSAR, and both docking-first three-stage variants) consistently exhibited poor precision and recall at every cutoff, indicating that the order in which methods are combined is at least as consequential as the choice of methods themselves, and that docking-first funnels offer no advantage at any experimental budget.

Taken together, these results show that, once compared under an identical experimental budget, elaborate integration is rarely necessary to achieve strong performance: a single well-validated predictor (ML-QSAR) or a minimal two-method rank fusion captures nearly all of the achievable hit recovery at a given screening depth, whereas most of the more elaborate strategies benchmarked here provide no measurable benefit. A Friedman rank-sum test on the per-target number of recovered actives rejected the hypothesis that the 20 strategies perform equivalently at each of the three budgets (χ^2^(19) = 81.0, 84.0, and 80.3 at the 1%, 5%, and 10% cutoffs; Monte Carlo permutation *p* < 1.0 × 10^−5^, 100,000 permutations, random seed = 42). Because no permutation produced a Friedman statistic greater than or equal to the observed value, the reported *p*-value corresponds to the minimum attainable empirical probability for the adopted number of permutations. This global result is driven predominantly by the wide separation between the ligand-based workflows and the docking-first funnels, and it should not be read as evidence that neighboring strategies differ from one another: with five blocks, the test can establish that the strategy set is heterogeneous, but it carries no information about any individual pairwise contrast. Post-hoc comparisons were therefore not attempted, since no signed-rank comparison over five paired observations can reach conventional significance irrespective of the effect size.

Consistent with this, the margin between the two leading strategies is small in absolute terms. At the 5% and 10% budgets, QSAR–MCS best-rank fusion recovered an average of 0.8 and 1.0 more actives out of 50 than standalone ML-QSAR (1.6 and 2.0 percentage points of recall), and the ordering was reversed for hERG at the 5% budget and for HIV1-P at the 10% budget. The two options are therefore best regarded as practically equivalent at the resolution of this benchmark, with the choice governed by data availability rather than measured performance: MCS querying requires a set of known actives large and structurally coherent enough to yield representative queries, which is also the source of the larger inter-target dispersion observed for all MCS-containing workflows.

Practically, standalone ML-QSAR is the natural choice when a very small, near-pure candidate list is required (≈1% of the library), where it is the only strategy that reaches the maximum attainable number of recovered actives for every target. For moderate-to-large screening budgets (5–10%), either standalone ML-QSAR or its best-rank fusion with MCS is a reasonable default, the latter offering a small average gain when suitable reference ligands are available. The three-method best-rank fusion remains a more conservative alternative when robustness across structurally diverse targets is prioritized over average performance. Conversely, the disadvantage of docking-first funnels is the one-ordering effect large enough to be stated with confidence on this panel. Given the limited five-target panel, all remaining comparisons should be regarded as descriptive and not interpreted as evidence of universal statistical superiority.

### 2.5. Target-Specific Behavior and Inter-Target Variability

Performance varied across biological targets, particularly for workflows incorporating molecular similarity, which reflects differences in ligand chemotype diversity and scaffold conservation. Under the fixed cutoff evaluation framework, these target-dependent effects remained evident, indicating that they arose from intrinsic target characteristics rather than differences in candidate list size. Among the individual methods, the ML-QSAR classifier exhibited the most consistent performance across all targets, with a standard deviation not exceeding 9.8 pp in recall at the 10% screening fraction. In contrast, MCS-based workflows displayed substantially greater variability, consistent with their dependence on the availability of structurally related reference ligands.

**Figure 5:**
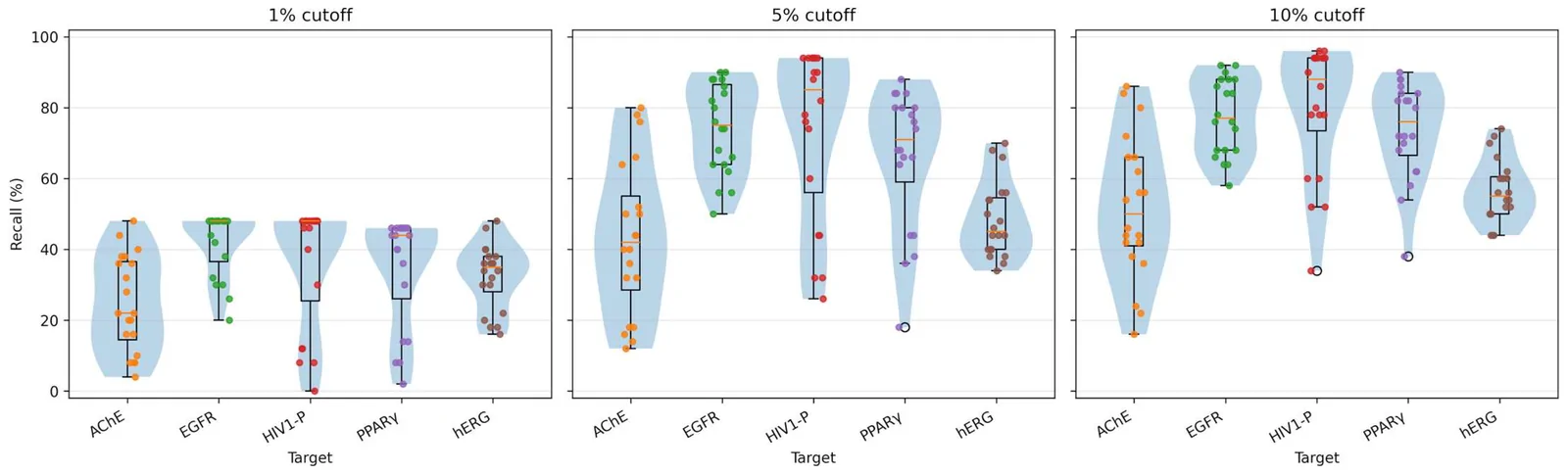
Distribution of strategy-level recall across the five benchmark targets at fixed screening fractions. For each screening fraction (1%, 5%, and 10%), violin/box-and-jitter plots summarize the recall values obtained by the 20 benchmarked strategies for AChE, EGFR, HIV1-P, PPARγ, and hERG. Each point represents one strategy. The plots highlight both the target dependence of screening performance and the variability among strategies within each target.

The representative target-level results further illustrate these trends. At the 1% screening fraction, the standalone QSAR recovered approximately 48% of the known actives and achieved 100% precision for four of the five targets. At the 5% fraction, strategies recovering a larger number of actives also showed proportionally higher precision because each workflow selected the same number of compounds. Accordingly, under the matched-budget design, target-specific differences should be interpreted primarily as differences in true-positive recovery, rather than as a purity–coverage trade-off. The complete target-level results are reported in Spreadsheet S1. This inter-target heterogeneity is summarized in Figure 5, which shows, for each screening fraction, the distribution of recall values across the 20 benchmarked strategies for the five targets.

Notably, hERG was included as a classification benchmark rather than a conventional therapeutic target. Because hERG blockade represents an undesirable off-target liability, the positive class corresponds to compounds that should be correctly identified and deprioritized. Consequently, recall and precision retain the same interpretation as for the therapeutic targets, but their practical objective is the reliable identification of potentially cardiotoxic compounds. In addition, a pChEMBL threshold of ≥6.0 (corresponding to IC_50_ ≤ 1 µM) was applied uniformly across all five targets to maintain a consistent, target-independent definition of active and inactive compounds. For hERG, this threshold was used solely as an operational classification criterion for benchmarking virtual screening strategies and should not be interpreted as a cardiac-liability or safety cutoff.

### 2.6. Chemical-Space and Structural-Similarity Characterization of Benchmark Datasets

Across all five benchmark targets, the physicochemical property distributions of the LUDe-generated decoys were broadly comparable to those of the active compounds across the evaluated descriptors, including MW, cLogP, TPSA, HBD, HBA, rotatable bonds, formal charge, FractionCSP3, and aromatic proportion (Figure S2). At the pooled level, standardized mean differences (SMDs) indicated generally limited separation between actives and decoys; however, target-specific analyses revealed moderate residual imbalances for some descriptors (Spreadsheet S1: SMD_summary tab), indicating that physicochemical matching was overall satisfactory but not uniform across all targets and properties.

**Figure 6:**
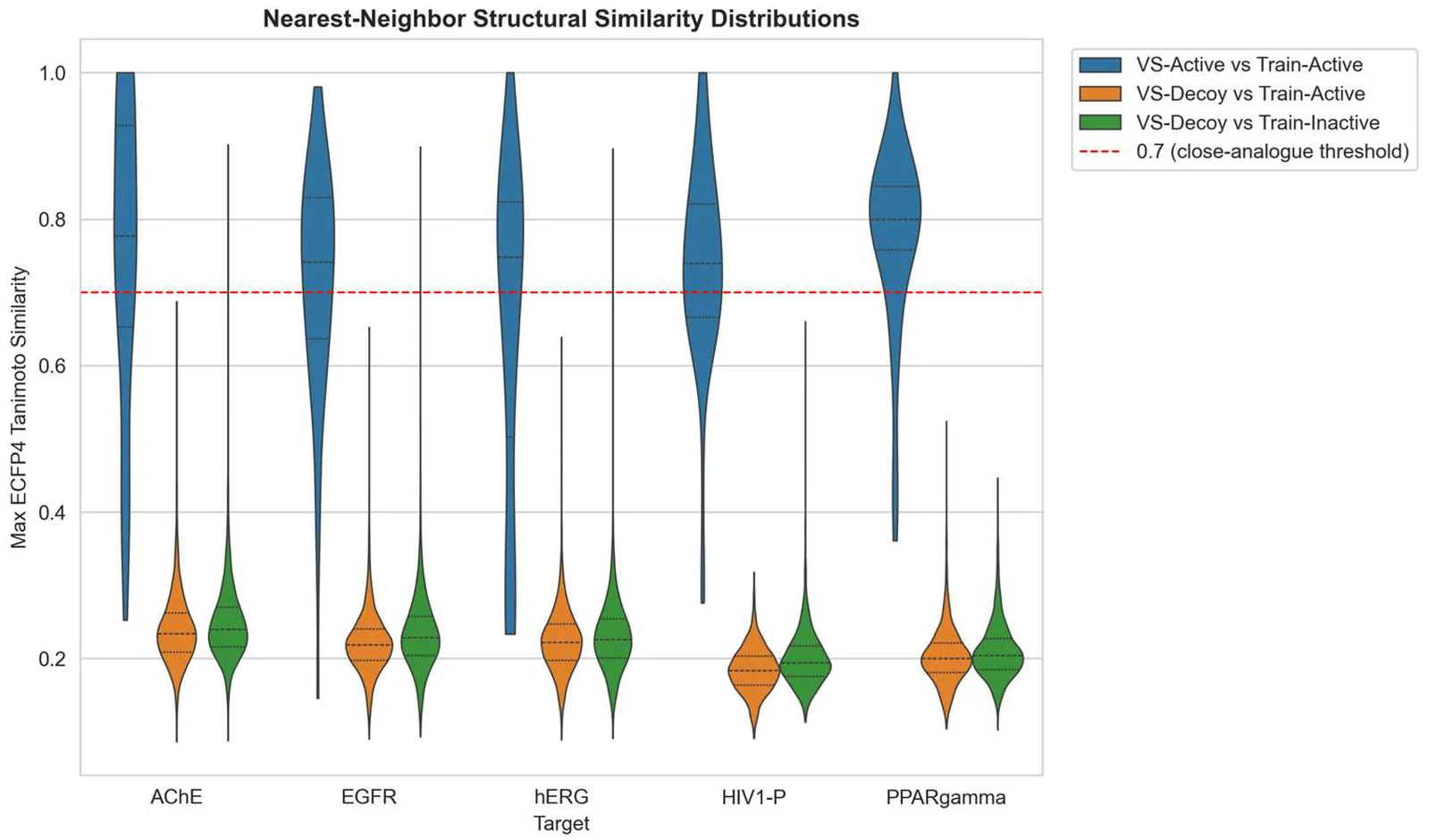
Nearest-neighbor structural similarity distributions between virtual screening datasets and training sets. Violin plots representing the distribution of maximum ECFP4 Tanimoto similarity scores (*T_c_*) calculated against the training sets across five targets (AChE, EGFR, hERG, HIV1-P, and PPARγ). Blue violins illustrate the similarity of active virtual screening compounds to training active molecules (VS-Active vs. Train-Active), highlighting high structural proximity within the applicability domain. Orange and green violins depict the maximum similarity of LUDe-generated decoys relative to training actives (VS-Decoy vs. Train-Active) and training inactives (VS-Decoy vs. Train-Inactive), respectively. The horizontal red dashed line marks the *T_c_* = 0.7 threshold commonly used to identify close structural analogues.

VS active compounds showed substantial structural proximity to known training actives, displaying a median Tanimoto similarity scores (*T_c_*) > 0.7 across targets (Figure 6). Specifically, 54% to 80% of VS active compounds possessed a *T_c_* ≥ 0.7 relative to the training set active compounds, and 98.0% fell strictly within the ML-QSAR applicability domain. Conversely, LUDe-generated decoys showed consistently low similarity to both training actives and training inactives (median *T_c_* ≈ 0.2, with >90% of decoys displaying *T_c_* < 0.3), demonstrating that the decoy background is topologically distant from the known active chemical space. Although physicochemical property matching was generally preserved, this topological separation may facilitate discrimination by ligand-based methods and therefore represents an important source of optimism in the retrospective benchmark. These findings demonstrate that the benchmark active class contains a substantial analog-rich component, providing an essential context for interpreting the performance of ligand-based methods.

### 2.7. Bemis–Murcko Scaffold Diversity and Training-Set Similarity Stratification

To evaluate the structural novelty and diversity of the retrieved active space, we performed a Bemis–Murcko scaffold analysis of the 50 prospective VS active compounds and decoy sets across all five benchmark targets, followed by a direct topological comparison with the training-set active scaffolds.

Across all targets, the 50 VS actives displayed exceptional structural diversity (Spreadsheet S1). The Scaffold-to-Compound Ratio (SCR) ranged from 0.80 for PPARγ (40 unique scaffolds among 50 compounds) to 0.98 for HIV1-P (49 unique scaffolds among 50 compounds), with AChE, EGFR, and hERG each exhibiting an SCR of 0.90 (45 unique scaffolds). The majority of identified scaffolds were singletons, ranging from 34 singletons in PPARγ to 48 in HIV1-P, with maximum cluster sizes restricted to just 2–4 compounds per scaffold and average compound-per-scaffold ratios between 1.02 and 1.25. Furthermore, scaffold overlap between VS actives and VS decoys was negligible across all targets, with Jaccard overlap coefficients ≤ 0.00089 (exactly 0.0000 for EGFR and HIV1-P), confirming that the decoy chemical space is structurally distinct from active scaffold topologies.

#### Differential Retrieval of Lower- and Higher-Similarity Actives

To investigate whether VS methods exhibited a systematic dependence on structural similarity to established chemical series, active compounds were stratified according to their nearest-neighbor similarity to training-set actives. For the comparative analysis, the lower-similarity stratum comprised 85 compounds, whereas the higher-similarity stratum comprised 80 compounds, representing, respectively, the least and most similar actives within the target-specific similarity distributions. Statistical differences in recall between these two strata were evaluated using Fisher’s exact test at selection budgets of 1.0%, 5.0%, and 10.0% (Table 2).

Ligand-based approaches demonstrated a strong preference for higher-similarity actives. ML-QSAR displayed the most pronounced similarity-dependent enrichment. At a 5.0% cutoff, ML-QSAR achieved near-saturated recovery in the higher-similarity stratum (97.5%, 78/80), whereas recall decreased to 54.1% (46/85) in the lower-similarity stratum (OR = 0.030, *p* = 7.94 × 10^−12^). This disparity was even more pronounced at the 1.0% cutoff, where ML-QSAR recovered 81.2% of higher-similarity actives compared with 16.5% of lower-similarity actives (*p* = 1.19 × 10^−17^). Similarly, MCS showed a statistically significant preference for higher-similarity actives across all selection thresholds (*p* < 0.002), reaching 78.8% recall in the higher-similarity stratum versus 52.9% in the lower-similarity stratum at a 5.0% budget (OR = 0.304). These findings demonstrate that 2D fingerprint-based and substructure-based methods predominantly recover compounds structurally related to known active series. In contrast, docking showed a substantially weaker dependence on similarity to training-set actives. No significant difference between the lower- and higher-similarity strata was observed at the 5% and 10% budgets, whereas a small-count difference was detected at the 1% cutoff. At the 5.0% selection cutoff, Docking yielded virtually identical recall rates across both strata: 23.5% (20/85) for lower-similarity actives and 21.2% (17/80) for higher-similarity actives (OR = 1.140, *p* = 0.852). This equitable distribution persisted at the 10.0% cutoff (31.8% vs. 35.0%, OR = 0.865, *p* = 0.742).

Furthermore, we analyzed the SCR, singleton fraction, and maximum cluster size of the selected compounds across the five benchmark targets and screening budgets. At the narrowest selection budget (1.0%), corresponding to the most restrictive prioritization setting, structure-based and sequential workflows generally retained greater scaffold diversity than standalone ligand-based approaches (Table 3).

Single docking yielded a high mean SCR of 0.942 ± 0.063, with 96.4% of selected compounds corresponding to singleton scaffolds. In comparison, Single MCS showed lower scaffold diversity (SCR = 0.832 ± 0.110; 84.1% singletons), consistent with its emphasis on shared substructural similarity. Single QSAR showed an intermediate profile (SCR = 0.874 ± 0.082; 86.8% singletons). These results indicate that the chemical diversity of the prioritized set depends not only on the absolute number of recovered active compounds but also on the nature of the structural information driving the ranking.

The effect was particularly evident for sequential workflows, in which the first-stage filter dictates the chemical space available to subsequent evaluation stages. Sequential (Docking -> QSAR) produced the highest mean scaffold diversity among all evaluated workflows (SCR = 0.950 ± 0.036; 94.6% singletons), accompanied by a low maximum cluster size (1.8 compounds per scaffold). Similarly, workflows in which docking was applied at the initial filtering stage consistently retained greater scaffold diversity than workflows using QSAR or MCS as the primary filter (SCR = 0.840–0.849), demonstrating that early ligand-based filtering can restrict downstream structural diversity.

Consensus and rank-fusion strategies provided a balanced intermediate profile between the standalone and sequential approaches. Consensus (Mean Rank 3M) maintained high scaffold diversity at the 1.0% cutoff (SCR = 0.899 ± 0.088) and expanded further at broader selection budgets (SCR = 0.957–0.959 at 5.0%). These results indicate that combining complementary ranking signals preserves scaffold breadth while effectively merging structure-, ligand-, and substructure-based scoring.

Importantly, scaffold diversity was evaluated directly on the compounds prioritized by each screening strategy rather than being inferred from the baseline composition of the benchmark library. Therefore, differences in SCR and singleton fraction directly reflect the intrinsic prioritization biases inherent to each computational workflow.

### 2.8. Practical Implications for Reproducible VS

Within the present retrospective benchmark, ML-QSAR showed the strongest standalone performance in terms of active recovery, particularly at the tightest screening budget. However, the similarity-stratified analysis demonstrated that this advantage was strongly dependent on the structural proximity of screened actives to the training set, highlighting an important limitation when extrapolating these findings to prospective campaigns. At the 1% budget, standalone ML-QSAR yielded the highest precision among the individual methods (99.2%), reflecting its strong enrichment of known-like actives within the fixed candidate list. Because precision and recall are algebraically linked under the fixed-budget design, the corresponding ranking of methods is identical for the two metrics; therefore, recall was used as the primary comparative outcome, while precision is reported for completeness.

Standalone docking consistently exhibited lower absolute active recovery than ligand-based approaches across all screening fractions, indicating that its main utility in this benchmark lies in providing complementary structural information within hybrid workflows rather than operating as a standalone primary screening method. Nevertheless, whereas ligand-based methods show substantial performance decay as structural similarity to the training set decreases, docking exhibits a substantially weaker dependence on training-set similarity, particularly at the 5% and 10% screening fractions.

These findings support a decision-oriented approach to virtual screening, where computational strategies are tailored to the specific goals and experimental capacity of a discovery campaign:

(i) For chemical-space exploration, docking-first or consensus workflows may be considered when preserving structural diversity within the prioritized candidate set is an explicit secondary objective. In the present benchmark, this increased candidate-list diversity was accompanied by lower active recovery; therefore, it should not be interpreted as direct evidence of superior novel-active discovery.
(ii) For Hit-to-Lead & series expansion, employ QSAR- or MCS-first funnels, which intentionally focus selection on dense congeneric clusters optimized for rapid SAR exploration and chemical analog optimization.

The present results also emphasize that individual VS methods should be viewed as sequential decision stages rather than as interchangeable scoring modules. In a multistage workflow, each additional step should justify its inclusion by providing incremental information or improving the downstream decision relative to the compounds surviving the preceding stage. Accordingly, pipeline order should not be determined solely by standalone recovery, but should also account for method complementarity, computational cost, and the type of output required by subsequent stages. For example, ligand-based ML-QSAR or similarity methods can efficiently prioritize compounds but do not generate protein–ligand binding poses; when downstream structural refinement by molecular dynamics, free-energy calculations, or interaction analysis is required, a pose-generation step such as molecular docking remains necessary even if docking is not the strongest initial ranking method. Thus, effective virtual high-troughput screening (VHTS) design requires both stage-wise performance validation and explicit consideration of how the output of one method is transferred to the next [20].

### 2.9. Limitations and Future Perspectives

This study had several limitations that should be considered when interpreting our results. First, the benchmark was performed on retrospective datasets with experimentally validated actives and property-matched decoys generated using LUDe. Although LUDe decoys were generated to approximate the physicochemical-property distributions of the active compounds, target-specific analyses revealed residual imbalances for some descriptors. More importantly, the decoys remained topologically distant from the active chemical space (median *T_c_* ≈ 0.2) and showed negligible overlap with the core scaffolds represented among the active series. Because experimentally inactive compounds encountered in prospective screening campaigns may include close structural analogues and activity cliffs (*T_c_* ≥ 0.7), discrimination between actives and topologically distant decoys may be comparatively easier for 2D ligand-based methods such as ML-QSAR and MCS. Consequently, the early-enrichment performance observed for these methods in the present retrospective benchmark should be regarded as potentially optimistic relative to that expected in real-world prospective screening campaigns. More generally, decoy-generation procedures may introduce latent structural patterns that are not fully captured by conventional physicochemical-property matching and that can potentially be exploited by machine-learning classifiers. The marked topological separation observed here between LUDe-generated decoys and the active chemical space indicates that such benchmark-specific effects cannot be excluded. Validation on prospective libraries or harder, decoy-independent benchmarks will be important to confirm the generality of the proposed workflows. Second, docking was intentionally evaluated under default, non-optimized conditions to maximize the reproducibility of the benchmark. While this approach provides a realistic baseline for integration strategies, it may also underestimate the true performance achievable with structure-based virtual screening. The success of molecular docking is highly dependent on multiple factors, including protein structure quality, binding site definition, conformational sampling, and the choice of scoring functions. In addition, the use of a single receptor structure per target cannot capture the full conformational heterogeneity of the binding site, and alternative crystallographic or cryo-EM structures could therefore yield different enrichment profiles. Target-specific optimization—such as refining ligand protonation states, adjusting metal-coordination parameters [21], or accounting for receptor flexibility—could substantially improve structure-based predictions. Consequently, the conclusion that docking-first workflows underperform must be interpreted with caution; this observation strictly reflects the standardized protocol applied in this benchmark and may not hold for docking campaigns that have undergone rigorous, target-specific tuning. Third, MCS similarity depends on the availability and diversity of known active compounds, which limits its applicability to poorly characterized targets. Fourth, only five protein targets were examined; because the per-target dispersion was large, the comparison of strategies would benefit from a broader target panel and formal statistical analysis to establish the generality and significance of the observed differences. The similarity-stratified analysis provides critical mechanistic context for these limitations, defining the operational boundaries of ligand- and structure-based methods. Because ML-QSAR and MCS rely on mapping prospective candidate molecules against known active space (via decision boundaries over ECFP4 fingerprints and maximum common substructure alignment, respectively), their performance advantage over docking is heavily concentrated among close structural analogs of training actives. In contrast, structure-based docking evaluates candidates via 3D physical and geometric complementarity within the binding pocket, making its retrieval behavior substantially less dependent on structural similarity to previously reported ligands.

Because all fixed-budget strategies selected the same number of compounds, the precision and recall were algebraically coupled within each target and cutoff. Therefore, the matched-budget results should be interpreted primarily as differences in hit recovery at equal assay capacity; variable-output set operations would instead be required to characterize a genuine recall–precision frontier.

Finally, an aspect not explicitly addressed here is the integration of multi-parameter optimization criteria, including ADMET properties and structural liability filters such as PAINS, which are routinely applied during lead identification and could be combined with the screening strategies evaluated here. Future studies should investigate how coupling activity prediction with developability criteria affects both enrichment and decision-making in prospective VS campaigns.

## 3. Materials and Methods

### 3.1. Data Retrieval and Curation

Datasets for all targets were retrieved from the ChEMBL database (release 36; accessed on 30 April 2026). Accordingly, the active compounds used in this study were derived directly from curated ChEMBL bioactivity records rather than from preassembled virtual-screening benchmark collections such as DUD-E or LIT-PCBA. The same data collection and curation workflows were applied to each target using RDKit (v2022.09.5; RDKit: Open-source cheminformatics, https://www.rdkit.org) and Python (v3.11). Compounds were selected by querying the corresponding UniProt identifiers (Table S2) and retaining single-protein assays that reported the IC_50_ values in the literature.

Raw structures were standardized by removing duplicates, incomplete records, and entries without corresponding pChEMBL values. For compounds with multiple activity measurements, structures showing discrepancies greater than 1.2 log units (ΔpChEMBL > 1.2) were discarded, and the median pChEMBL value was assigned to the remaining structures. Although these filtering and aggregation criteria reduce the impact of cross-assay variability, residual heterogeneity arising from differences in experimental protocols and assay conditions cannot be completely excluded and represents an inherent limitation of retrospective bioactivity datasets [22]. Salts, counterions, and small fragments were removed, the structures were normalized and neutralized, and canonical tautomers were generated using the RDKit rdMolStandardize module (v2022.09.5; RDKit: Open-source cheminformatics, https://www.rdkit.org).

The compounds were classified as active (pChEMBL ≥ 6.0) or inactive (pChEMBL < 6.0). To generate datasets for model development and VS, 50 active compounds were selected using scaffold-stratified sampling and used to generate decoys with LUDe (v1.0; https://github.com/LIDeB/LUDe.v1.0, accessed on 11 May 2026) [23] at a 1:50 active-to-decoy ratio, yielding the VS dataset. Because LUDe did not return the full complement of decoys for every active and a small number of structures failed fingerprint or 3D-coordinate generation, the final VS libraries contained 2345–2549 compounds, each retaining all 50 confirmed actives.

The remaining compounds were divided into training (80%) and test (20%) sets using scaffold- and activity-stratified sampling methods, respectively. The training set was balanced to a 1:1 active/true inactive ratio, whereas the external test set retained the remaining compounds in the dataset. The training and test sets were generated using scaffold-based splitting, combined with activity stratification. Training and test sets were used for model development and validation, and the VS dataset was employed to evaluate the prospective screening performance using enrichment metrics. To prevent data leakage, the compounds present in the VS dataset were excluded from the training dataset prior to model construction. Although the VS database includes synthetic decoys to reproduce the low prevalence of active compounds encountered in prospective screening campaigns, both model development and independent test-set evaluation were performed exclusively on experimentally annotated active and inactive compounds. Consequently, decoys were used only to reproduce realistic screening conditions, rather than to train or validate the predictive models.

### 3.2. Machine-Learning QSAR (ML-QSAR)

#### 3.2.1. Molecular Featurization

Standardized canonical SMILES were converted into Extended Connectivity Fingerprints (ECFP4) [24] using RDKit (radius = 2, 2048 bits), with chirality information included. Molecules that failed fingerprint generation were excluded from further analysis.

#### 3.2.2. Model Training, Calibration, and Validation

A random forest (RF) classifier was implemented using scikit-learn (v1.7.2) [25] with 500 decision trees, balanced class weights, and max_features set to the square root of the total number of descriptors. To obtain calibrated class probabilities, the RF model was combined with Platt scaling through CalibratedClassifierCV using sigmoid calibration.

The model performance was assessed using stratified 5-fold cross-validation, which preserved the class distribution across the folds. The final calibrated model was retrained using the complete training set.

Random Forest was selected as a robust and computationally efficient baseline for fingerprint-based classification, particularly suited to moderate-sized medicinal-chemistry datasets represented by sparse ECFP descriptors, as previously observed in other studies [19]. The objective of this study was not to benchmark alternative machine-learning architectures, but to compare integration strategies under matched experimental budgets using a well-established and reproducible ligand-based predictor. More complex architectures, including gradient boosting, graph neural networks, and transformer-based models, may provide additional gains and represent an important direction for future benchmarking.

#### 3.2.3. Performance Evaluation and Bootstrap Analysis

The predictive performance was evaluated using the ROC-AUC, PR-AUC, Brier score, precision, recall, F1-score, Matthews Correlation Coefficient (MCC), and Enrichment Factors at the top 1%, 5%, and 10% of ranked compounds (EF1%, EF5%, and EF10%). A probability threshold of 0.5 was used for the binary classification.

Metric robustness was estimated using bootstrap resampling (100 iterations), with 95% confidence intervals calculated from the 2.5th and 97.5th percentiles of the bootstrap distributions.

#### 3.2.4. Applicability Domain Definition and VS Application

The applicability domain (AD) was defined using a principal component analysis (PCA)-based Mahalanobis distance approach in a reduced feature space. The AD boundary was set to the 95th percentile of the training distance distribution. During VS, compounds falling within this boundary were considered inside the AD and classified using the calibrated RF model, whereas compounds outside the AD were flagged as lower-confidence predictions. Approximately 98% of the screened active compounds and 99.94% of the LUDe-generated decoys fell within the defined applicability domain, indicating that the AD criterion did not substantially restrict either class within the virtual screening space. As AD characterization was not a primary objective of the present study, the corresponding results are reported in Figure S3.

### 3.3. Maximum Common Substructure (MCS) Similarity

#### 3.3.1. Query Selection

Active compounds in the training set were clustered using the Butina algorithm [26] based on Morgan fingerprint (radius = 2) and Tanimoto distances (cutoff = 0.4). Clusters containing at least two molecules were retained, and the centroids of the ten largest clusters were selected as representative structural queries. Up to ten candidate queries were evaluated for each target, depending on the number of valid clusters obtained after clustering. The query yielding the highest ROC-AUC on the validation set was selected as the best query. No ties in ROC-AUC occurred during the best-query selection.

#### 3.3.2. Similarity Scoring and Threshold Optimization

Maximum common substructures (MCS) were computed between each query and target molecule under topological constraints, requiring the atom type, bond order, and ring correspondence. Structural similarity was quantified using a normalized score combining atom and bond conservation (Equation (1)):

(1) $$Similarity=0.5\;\times \;\left(\frac{A_{common}}{A_{q}+\;A_{t\;}-\;A_{common}}\right)\;+\;0.5\;\times \;\left(\frac{B_{common}}{B_{q}+\;B_{t\;}-\;B_{common}}\right),$$

where *A_common_* and *B_common_* are the number of atoms and bonds in the maximum common substructure, *A_q_* and *B_q_* those of the query molecule, and *A_t_* and *B_t_* those of the screened molecule. Equal weighting of the atom- and bond-conservation terms was adopted to avoid introducing an a priori preference for either molecular size overlap or connectivity preservation. The weighting was fixed before the model evaluation and was not optimized on the virtual screening benchmark.

For each compound, similarity was calculated against all query structures, and a consensus score was defined as the maximum similarity obtained. Optimal classification thresholds were determined on the validation set by maximizing Youden’s index on the ROC curve. The query achieving the highest ROC-AUC was selected as the best query. Final performance was assessed on a fully independent VS set.

#### 3.3.3. MCS Virtual Screening

The optimized thresholds, Best Query, and consensus approach were applied to the VS library. Performance was evaluated using ROC-AUC, BEDROC (α = 20), EF1%, EF5%, EF10%, precision, and recall to assess both ranking ability and classification performance.

### 3.4. Molecular Docking Analysis

#### 3.4.1. Ligand Preparation

The 3D structures of the VS dataset were generated and minimized using Open Babel (v3.1.1) [27]. Hydrogens were added to reflect physiological pH (7.4) states, and the obtained ligands were minimized using the Merck Molecular Force Field 94 (MMFF94).

#### 3.4.2. Protein Preparation

For each target, a ligand-bound experimental structure was selected to provide a structurally defined binding site and a co-crystallized ligand suitable for redocking validation. When multiple structures were available, preference was given to entries providing an appropriate representation of the pharmacologically relevant binding site and sufficient structural quality for reproducible docking calculations (Table S2). The protein was prepared using YASARA software (v25.1.13, YASARA Biosciences GmbH, Vienna, Austria) [28] by adding hydrogens using the “Clean → All” option. Finally, the co-crystallized ligand and water molecules were removed using YASARA software. The protein models were validated by redocking the co-crystallized ligand; the docking algorithm demonstrated the ability to reproduce the co-crystallized conformation. The heavy-atom RMSD was calculated using the “obrms” Open Babel tool. Four of the five systems yielded redocking RMSD values ≤ 2.0 Å, whereas AChE yielded a value of 2.06 Å (Table S3).

#### 3.4.3. Molecular Docking

Docking evaluations were performed using the GNINA algorithm (v1.3.2) [29]. GNINA was selected based on a previous comparative benchmark in which its CNN-rescored poses outperformed AutoDock Vina across ten structurally heterogeneous targets [30]. Ligand poses were generated with an exhaustiveness of 8, producing up to nine poses for each ligand. The poses were then processed using the tool’s “rescore” functionality (--cnn_scoring rescore). Specifically, the default CNN model was configured to evaluate 15 grid rotations for each generated pose (--cnn_rotation 15), using the CNN score to rank the outputs. The input files were formatted as PDB files for the proteins and SDF files for the ligands. To establish the simulation environment, a bounding box was constructed around the reference co-crystallized ligand using AutoDock Tools (v1.5.7) [31]. The grid boundaries were set generously wide to prevent any artificial forcing of binding geometries. A uniform grid spacing of 1.0 Å was applied to all targets. Specifically, the grid centers (x, y, z) and box dimensions (x, y, z, in Å) were defined as follows: AChE (center: 90.398, 94.750, 13.486; dimensions: 29, 27, 26), EGFR (center: 22.030, 0.300, 51.550; dimensions: 23, 34, 27), HIV1-P (center: 20.208, −2.781, 20.425; dimensions: 31, 29, 26), PPARγ (center: 16.875, 7.580, 44.931; dimensions: 24, 20, 24), and hERG (center: 157.033, 151.298, 132.491; dimensions: 22, 22, 30). For complete reproducibility, the automatically generated random seeds for each run were −1670277022 (AChE), 1786979296 (EGFR), 768701600 (HIV1-P), −1450763440 (PPARγ), and 1893347422 (hERG). Molecular docking was executed using the following general GNINA command line: ‘gnina -r protein.pdb -l ligands.sdf --center_x [X] --center_y [Y] --center_z [Z] --size_x [X] --size_y [Y] --size_z [Z] --cnn_scoring rescore --cnn_rotation 15 --log log.txt -o docked.pdb’.

To perform the sensitivity analysis on the AChE target, two additional docking configurations were evaluated: one maintaining the default grid box but increasing the exhaustiveness to 32 (Protocol B) and another combining an exhaustiveness of 32 with a focused bounding box centered precisely on the co-crystallized ligand (Protocol C; center x, y, z: 94.898, 94.750, 16.986; dimensions: 21, 15, 20 Å). The TP and recall metrics for these variations were calculated using the identical fixed-budget cutoffs applied to the main benchmark.

### 3.5. Integration Strategies and Screening-Fraction Evaluation

Each compound *i* in a virtual screening library containing *N* compounds was characterized by three independent scores obtained as described above: the ML-QSAR active-class probability, docking CNN score, and MCS consensus similarity, all oriented so that larger values indicate higher priority. For each screening fraction *f* (*f* = 0.01, 0.05, and 0.10), an identical experimental budget was imposed across all strategies by selecting exactly *k* = ⌊*f*·*N*⌋ compounds. The 1%, 5%, and 10% fractions were selected to represent progressively less restrictive experimental follow-up scenarios, ranging from highly selective compound prioritization to moderate and broader assay capacity. Because the monetary cost of experimental validation is strongly assay- and target-dependent, no fixed financial value was assigned to these fractions; throughout this study, the budget therefore denotes the number of compounds advanced to experimental testing. Consequently, every workflow prioritized the same number of candidates, allowing direct comparison of recall and precision independently of candidate list size.

Twenty screening strategies were evaluated and grouped into five methodological categories.

Individual methods. Each method ranked the complete library according to its score, and the top-*k* compounds were selected.

Because every workflow was required to return exactly *k* compounds, the combination operators used here are rank fusion analogs rather than literal set union and set intersection. Therefore, we refer to the minimum-rank operator as best-rank (OR-like) fusion and the maximum-rank operator as worst-rank (AND-like) fusion.

Parallel integration (best rank fusion). To emulate the behavior of a union strategy while maintaining a fixed screening budget, each compound was assigned the best (minimum) rank obtained among the constituent methods (Equation (2)):

(2) $$R_{i}^{union}\;=\min(r_{i}^{\left(1\right)},r_{i}^{\left(2\right)},\ldots )$$

where $r_{i}^{\left(m\right)}$ denotes the rank assigned to compound *i* by method *m* (rank 1 = highest priority). Compounds were then ordered according to $R_{i}^{union}$, and the top-*k* candidates were retained. This aggregation favors compounds highly ranked by at least one constituent method, reproducing the exploratory behavior of a conventional union strategy while preserving an identical experimental budget.

Intersection (worst-rank fusion). To emulate intersection-based prioritization, each compound received the worst (maximum) rank among the constituent methods (Equation (3)):

(3) $$R_{i}^{intersection}\;=\max(r_{i}^{\left(1\right)},r_{i}^{\left(2\right)},\ldots )$$

Compounds were subsequently ranked according to $R_{i}^{intersection}$, and the top-*k* candidates were selected. This procedure preferentially retains compounds consistently ranked highly by all participating methods, while ensuring that every strategy returns the same number of compounds.

Rank-based consensus. Consensus ranking was obtained by averaging the individual method ranks (Equation (4)):

(4) $$R_{i}\;=\;\frac{1}{3}\left(r_{i}^{QSAR}\;+\;r_{i}^{MCS}\;+\;r_{i}^{Dock}\right),$$

where $r_{i}^{QSAR},\;r_{i}^{MCS},\;r_{i}^{Dock}$ are the ranks assigned by the individual methods. Compounds were sorted according to the consensus rank (ascending order), and the top-*k* candidates were retained.

Sequential funneling. Sequential workflows were implemented as true multi-stage filtering procedures. In a two-stage funnel, A → B, the first method reduced the library to an intermediate subset larger than the final screening budget (equal to 2*k* compounds). The surviving compounds were then re-ranked exclusively according to the second method, and the final top-*k* candidates were selected from this reduced set.

Three-stage workflows followed the same principle. The first stage retained the top 3*k* compounds, the second stage further reduced the candidate pool to 2*k*, and the third stage performed a final re-ranking to obtain the final top-*k* compounds. All six possible two-stage and all six possible three-stage orderings of QSAR, MCS, and docking were evaluated.

Performance metrics. For a selected set *S* containing *a_S_* experimentally active compounds out of *A* known actives in the benchmark dataset, recall and precision were defined as follows (Equation (5)):

(5) $$Recall=\;\frac{a_{s}}{A},$$

and (Equation (6))

(6) $$Precision=\frac{a_{s}}{|S|},$$

respectively.

For every target and screening fraction, all 20 strategies were evaluated under the same experimental budget. Results are reported as the mean ± standard deviation across the five benchmark targets.

### 3.6. Statistical Analysis

Statistical differences among screening strategies were assessed using the Friedman test, considering benchmark targets as blocks and screening strategies as treatments. Because only five benchmark targets were available, the asymptotic χ^2^ approximation was not used to estimate statistical significance. Instead, *p*-values were estimated using a Monte Carlo permutation procedure (100,000 permutations; random seed = 42), while retaining the Friedman χ^2^ statistic as the omnibus test statistic. For each permutation, the strategy labels were randomly reassigned within each benchmark target, thereby preserving the block structure under the null hypothesis. The empirical permutation *p*-value was calculated as follows in Equation (7):

(7) $$p=\;\frac{1\;+\;\#(T_{perm\;}\geq {\;T}_{obs})}{1\;+\;N_{perm}},$$

where $T_{obs\;}$ is the observed Friedman statistic, $T_{perm}$ is the statistic obtained for each permutation, and $N_{perm}$ = 100,000. Calculations were performed with SciPy [32].

### 3.7. Scaffold Analysis

To quantify scaffold diversity within selected VS subsets, the Scaffold-to-Compound Ratio (SCR) was defined as (Equation (8)):

(8) $$SCR=\;\frac{N_{unique}}{N_{selected}},$$

where *N_unique_* is the number of unique Bemis–Murcko scaffolds and *N_selected_* is the total number of selected compounds within a given screening budget B. Additional scaffold parameters include the singleton fraction (the proportion of unique scaffolds represented by exactly one molecule) and the maximum compound frequency per scaffold.

To evaluate the structural convergence across virtual screening strategies, pairwise scaffold overlap was evaluated between sets of scaffolds *A* and *B* retrieved by different strategies at cutoffs of 1.0%, 5.0%, and 10.0%. Scaffold overlap was quantified using the Jaccard scaffold similarity index (*J*) as follows:

(9) $$J\left(A,B\right)=\;\frac{\left|A\cap B\right|}{\left|A\cup B\right|},$$

where ∣A∩B∣ represents the number of shared scaffolds and ∣A∪B∣ represents the total union of unique scaffolds retrieved by both strategies.

To investigate potential training-set analog bias, a similarity-stratified benchmark was performed. Extended-Connectivity Fingerprints (ECFP4 equivalent: Morgan fingerprints with radius r = 2 and 2048 bits) were generated for all 50 true VS active compounds and all training-set active compounds per target. For each VS active compound *i*, the maximum nearest-neighbor Tanimoto similarity (*T_c_*) against the set of training-set actives (T) was calculated using Equation (10):

(10) $$T_{c}\left(i\right)=\max Tanimoto({FP}_{i},\;{FP}_{j}),$$

where ${FP}_{i},\;{FP}_{j}$ are bit vectors representing the molecular fingerprints.

Within each target, VS active compounds were sorted deterministically by *T_c_* and partitioned into three target-specific terciles as follows:

- Low Similarity Bin (≈16–17 compounds per target)
- Mid Similarity Bin (≈16–17 compounds per target)
- High Similarity Bin (≈16–17 compounds per target)

Fixed-budget virtual screening selection was applied at fraction cutoffs of 1.0%, 5.0%, and 10.0%, where the compound selection budget was defined as *B* = (*N* × cutoff) for library size *N*. Active-compound recovery (Recall%) was calculated independently for each similarity tercile to quantify screening performance across different levels of structural similarity to training-set actives.

To evaluate the chemical novelty of the top-performing active compounds, Bemis–Murcko scaffolds of the top 50 active compounds per target (*S_VS_*) were compared with the scaffold space of the training-set active compounds (*S_Train_*). The scaffold space was partitioned as follows:

- Overlapping Scaffolds; *S_VS_*∩*S_Train_*
- Novel Scaffolds; *S_VS_*∖*S_Train_*

The percentage of novel scaffolds was determined as follows (Equation (11)):

(11) $$Novelty\;\left(\%\right)=\frac{\left|\frac{S_{VS}}{S_{Train}}\right|}{\left|S_{VS}\right|}\;\times \;100\;,$$

reflecting the capacity of each screening protocol to discover novel active frameworks that were not observed during model training.

Fisher’s Exact Test: Pooled counts of recovered versus non-recovered active compounds in the Low and High similarity terciles were compared using Fisher’s exact test on a 2 × 2 contingency matrix. Two-sided *p*-values and odds ratios (OR) were reported to evaluate analogue bias.

## 4. Conclusions

This study systematically benchmarked 20 virtual screening strategies integrating molecular docking, MCS similarity, and an ML-QSAR classifier under a standardized fixed-budget framework. By constraining every workflow to select the same number of compounds at each screening fraction, differences in performance reflected the intrinsic effectiveness of the prioritization strategy rather than variations in candidate-list size. Under these conditions, a clear hierarchy emerged. The standalone ML-QSAR classifier consistently achieved the highest across-target performance at the 1% screening fraction. The QSAR–MCS best-rank fusion achieved the highest across-target mean performance at the 5% and 10% screening fractions, although by a margin of approximately one active out of fifty over standalone ML-QSAR, whereas more elaborate integration schemes, including all consensus-based approaches, worst-rank fusion strategies, and sequential funnels, did not improve the average hit recovery observed across the benchmark. Target-level analyses further showed that the three-method best-rank fusion, although not achieving the highest average performance, remained competitive across individual targets and therefore represents a robust alternative when consistency across structurally diverse systems is preferred over maximizing average recall.

Among the individual screening methods, ML-QSAR was the most robust and accurate for all five targets. MCS similarity provided complementary information but was more dependent on the structural diversity of the known ligands. In contrast, when executed under the strictly default, non-optimized protocol employed here, standalone docking consistently produced lower active recovery than ligand-based approaches, yielding the lowest overall recall and precision. Crucially, similarity-stratified analysis revealed that the advantages of ligand-based methods were heavily driven by close structural analogs of the training active compounds (with high-similarity stratum recall reaching up to 97.5%). Despite its lower absolute recovery under these non-optimized baseline conditions, docking exhibited substantially weaker dependence on training-set structural similarity, particularly at the 5% and 10% budgets. Notably, using docking as the first stage of sequential workflows reduced overall hit recovery but preserved the highest scaffold diversity among the prioritized candidate sets (mean SCR = 0.950). This indicates that early docking filtering can preserve broader structural diversity among prioritized candidates, although this occurs at the cost of lower active recovery and should not be interpreted as evidence of superior novel-active discovery. From a practical perspective, the benchmark identifies three complementary decision scenarios. When only a very small fraction of the library can be experimentally tested (approximately 1%), standalone ML-QSAR offers the highest probability of recovering active compounds while maintaining an almost perfectly pure candidate list. For intermediate and larger screening campaigns (5–10%), QSAR–MCS best-rank fusion provides the best overall balance between hit recovery and candidate quality. Docking-first sequential funnels or consensus rank fusion may therefore be considered when preservation of candidate-list scaffold breadth is an explicit secondary objective, while recognizing their lower hit recovery under the evaluated protocol. When robustness across chemically heterogeneous targets is prioritized over maximizing average performance, the three-method best-rank fusion represents a conservative and reliable alternative.

Overall, these findings demonstrate that, once experimental budget is explicitly controlled, increasing methodological complexity does not necessarily translate into better virtual screening performance. Instead, a well-validated ligand-based predictor, either alone or combined with a simple complementary similarity measure, was sufficient to achieve the highest overall efficiency across the benchmark targets evaluated in this study. However, selecting the optimal strategy requires matching the workflow mechanism (topological analog retrieval vs. physical pocket binding) to the primary discovery objective (analog expansion vs. chemical space exploration). Therefore, this benchmark provides practical guidance for selecting virtual screening workflows according to available experimental resources while emphasizing the importance of evaluating computational strategies under realistic decision-making constraints.

Future work should extend this framework through prospective experimental validation, evaluation of larger and more chemically diverse libraries, and benchmarking against more challenging decoy-independent datasets. Incorporating additional decision criteria, such as ADMET properties, synthetic accessibility, and developability metrics, will further improve the relevance of decision-oriented virtual screening pipelines in early-stage drug discovery.

## Figures and Tables

**Table 1:** Mean recall and precision (±SD across five targets: AChE, EGFR, HIV1-P, PPARγ, and hERG) for the three individual scoring methods at 1%, 5%, and 10% database fraction cutoffs. SD, standard deviation, in percentage points (pp).

Method	Cutoff	Recall (%) ± SD	Precision (%) ± SD
ML-QSAR	1%	47.6 ± 0.9	99.2 ± 1.8
MCS	1%	38.8 ± 13.7	81.1 ± 29.3
Docking	1%	8.4 ± 9.0	17.3 ± 18.4
ML-QSAR	5%	81.6 ± 9.1	33.2 ± 4.2
MCS	5%	67.2 ± 24.6	27.4 ± 10.3
Docking	5%	28.0 ± 14.8	11.3 ± 5.9
ML-QSAR	10%	84.4 ± 9.8	17.2 ± 2.3
MCS	10%	72.8 ± 21.8	14.8 ± 4.6
Docking	10%	38.0 ± 15.3	7.7 ± 3.1

**Table 2:** Statistical evaluation of active-compound recovery across structural-similarity strata. Active compounds were stratified into lower-similarity (*N* = 85) and higher-similarity (*N* = 80) groups according to their nearest-neighbor ECFP4 similarity to training-set actives. Recall rates are presented as percentages with raw counts (*n*/*N*) across the 1.0%, 5.0%, and 10.0% selection fractions for docking, maximum common substructure (MCS), and machine-learning QSAR (ML-QSAR). Two-tailed Fisher’s exact tests were used to assess differential recovery between the two strata. An odds ratio (OR) < 1 indicates preferential recovery of higher-similarity actives.

Cutoff	Method	Low-Similarity Recall (%)	High-Similarity Recall (%)	Odds Ratio (OR)	Fisher *p*-Value
1.0%	Docking	1.2% (1/85)	8.8% (7/80)	0.124	3.03 × 10^−2^
	MCS	21.2% (18/85)	51.2% (41/80)	0.256	8.26 × 10^−5^
	ML-QSAR	16.5% (14/85)	81.2% (65/80)	0.046	1.19 × 10^−17^
5.0%	Docking	23.5% (20/85)	21.2% (17/80)	1.140	0.852 (ns)
	MCS	52.9% (45/85)	78.8% (63/80)	0.304	5.72 × 10^−4^
	ML-QSAR	54.1% (46/85)	97.5% (78/80)	0.030	7.94 × 10^−12^
10.0%	Docking	31.8% (27/85)	35.0% (28/80)	0.865	0.742 (ns) ^1^
	MCS	61.2% (52/85)	83.8% (67/80)	0.306	1.64 × 10^−3^
	ML-QSAR	62.3% (53/85)	97.5% (78/80)	0.042	4.17 × 10^−9^

^1^ ns: not significant at *p* > 0.05.

**Table 3:** Scaffold diversity of prioritized compounds at the 1.0% selection cutoff. Mean Scaffold-to-Compound Ratio (SCR) and singleton fraction are reported across the five benchmark targets. SCR values are presented as mean ± standard deviation (SD) across targets. The table highlights representative strategies from the major workflow categories; the complete results for all strategies and selection budgets are provided in Spreadsheet S1.

Strategy Category	Representative Strategy	Mean SCR ± SD	Singleton Fraction (%)
Structure-based sequential	Sequential (Docking → QSAR)	0.950 ± 0.036	94.6
Single method—structure-based	Single Docking	0.942 ± 0.063	96.4
Consensus/rank fusion	Consensus (Mean Rank 3M)	0.899 ± 0.088	91.1
Single method—ligand-based	Single QSAR	0.874 ± 0.082	86.8
Substructure-based	Single MCS	0.832 ± 0.110	84.1

## Data Availability

The original data presented in the study are openly available in the GitHub repository at https://github.com/elisabettatomarchio/beyond-the-score-vs-benchmark release v. 1.0.1, accessed on 11 August 2026 (archived at Zenodo, https://doi.org/10.5281/zenodo.21645344). The repository provides the analysis scripts together with the curated training, test and virtual-screening sets for all five targets, and the corresponding docking, MCS and ML-QSAR scores, allowing every result reported here to be regenerated. The code is released under the MIT License; the datasets, derived from ChEMBL, are redistributed under the Creative Commons Attribution-ShareAlike 3.0 Unported License.

## Supplementary Materials

The following supporting information can be downloaded at: https://www.mdpi.com/article/10.3390/ijms27167497/s1.

## Abbreviations

Abbreviations used in this manuscript are as follows:

AChE	Acetylcholinesterase
AD	Applicability domain
ADMET	Absorption, distribution, metabolism, excretion, and toxicity
AUC	Area under the curve
BEDROC	Boltzmann-enhanced discrimination of receiver operating characteristic
CADD	Computer-aided drug discovery
ChEMBL	Curated bioactivity database (EMBL-EBI)
CNN	Convolutional neural network
ECFP4	Extended-connectivity fingerprint (radius 2, 2048 bits)
EF	Enrichment factor (EF1%, EF5%, EF10%)
EGFR	Epidermal growth factor receptor
hERG	Human ether-à-go-go-related gene (potassium channel)
HIV1-P	Human immunodeficiency virus type 1 protease
MCS	Maximum common substructure
ML	Machine learning
ML-QSAR	Machine-learning QSAR classifier
PAINS	Pan-assay interference compounds
PCA	Principal component analysis
PDB	Protein Data Bank
pp	Percentage points
PPARγ	Peroxisome proliferator-activated receptor gamma
PR-AUC	Area under the precision–recall curve
QSAR	Quantitative structure–activity relationship
RF	Random forest
RMSD	Root-mean-square deviation
ROC-AUC	Area under the receiver operating characteristic curve
SD	Standard deviation
SDF	Structure-data file
SMILES	Simplified molecular-input line-entry system
UniProt	Universal Protein Resource
VS	Virtual screening
