# Supplementary material for: Beyond the Score: Fixed-Budget Benchmarking of Virtual Screening Integration Strategies for Decision-Centric Drug Discovery
Source: Int J Mol Sci. 2026 Aug 21;27(16):7497. doi: 10.3390/ijms27167497 (PMC13513552; doi:10.3390/ijms27167497)
Supplement: Supplementary file 1 [file ijms-27-07497-s001.zip › Beyond_the_Score_SI_IJMS_revised_2.pdf]

International Journal of
Molecular Sciences

MDPI

Supplementary Materials

Beyond the Score: Fixed-Budget Benchmarking of Virtual Screening Integration Strategies for Decision-Centric Drug Discovery

Elisabetta Grazia Tomarchio [1,2], Rocco Buccheri [1] and Antonio Rescifina [1,*]

[1] Department of Drug and Health Sciences, University of Catania, Viale A. Doria 6, 95125 Catania, Italy; elisabetta.tomarchio@phd.unict.it (E.G.T.); rocco.buccheri@unict.it (R.B.)
[2] Department of Biomedical and Biotechnological Sciences, University of Catania, Via Santa Sofia 97, 95123 Catania, Italy
* Correspondence: antonio.rescifina@unict.it

Table of contents

Table S1. Sensitivity analysis of the GNINA docking protocol on the AChE target dataset (2,483 total compounds, 50 expected actives). True Positives (TP) and Hit Recovery/Recall (%) are reported across three evaluation budgets. Protocol A represents the default conditions used throughout the benchmark.
Table S2. Access codes (UNIPROT, ChEMBL, and PDB codes, source, and chain used for docking analysis) of proteins used in this work.
Table S3. Redocking RMSD values of co-crystallized ligands.
Figure S1. Comparison of VS integration strategies under a fixed experimental budget. Mean precision values across all targets.
Figure S2. Physicochemical property of actives and decoys**.**
Figure S3. Applicability Domain (AD) analysis of all targets.
Spreadsheet S1. Per-compound virtual-screening scores, target-level fixed-budget results, aggregated statistics, and ML-QSAR/MCS validation metrics, and the quality-control report of the score-merging step (as a separate file: Beyond_the_Score_VS_results_SI_IJMS.xlsx).

Table S1. Sensitivity analysis of the GNINA docking protocol on the AChE target dataset (2,483 total compounds, 50 confirmed actives). True Positives (TP) and Hit Recovery/Recall (%) are reported across three evaluation budgets. Protocol A represents the default conditions used throughout the benchmark.

Budget Fraction	Protocol A (Default Box, Exh=8)	Protocol B (Default Box, Exh=32)	Protocol C (Focused Box, Exh=32)
1%	2 (4.0%)	3 (6.0%)	4 (8.0%)
5%	6 (12.0%)	6 (12.0%)	7 (14.0%)
10%	8 (16.0%)	11 (22.0%)	10 (20.0%)

Table S2. Access codes (UNIPROT, ChEMBL, and PDB codes, source, and chain used for docking analysis) of proteins used in this work.

Protein name	UNIPROT ID	ChEMBL ID	PDB ID	PDB source	Chain
AChE (Acetylcholinesterase)	P22303	CHEMBL220	6O4W	PDB-Redo	B
EGFR (Epidermal Growth Factor Receptor)	P00533	CHEMBL203	8SC7	PDB	A
HIV1-P (HIV-1 Protease)	Q72874	CHEMBL243	1XL2	PDB	A, B
PPARγ (Peroxisome Proliferator-Activated Receptor gamma)	P37231	CHEMBL235	4EMA	PDB	A
hERG (human Ether-à-go-go-Related Gene potassium channel)	Q12809	CHEMBL240	8ZYO	PDB	A–D

Table S3. Redocking RMSD values of co-crystallized ligands.

Protein Name	RMSD value (Å)
AChE	2.06
EGFR	1.97
HIV1-P	1.95
PPARγ	1.20
hERG	0.71

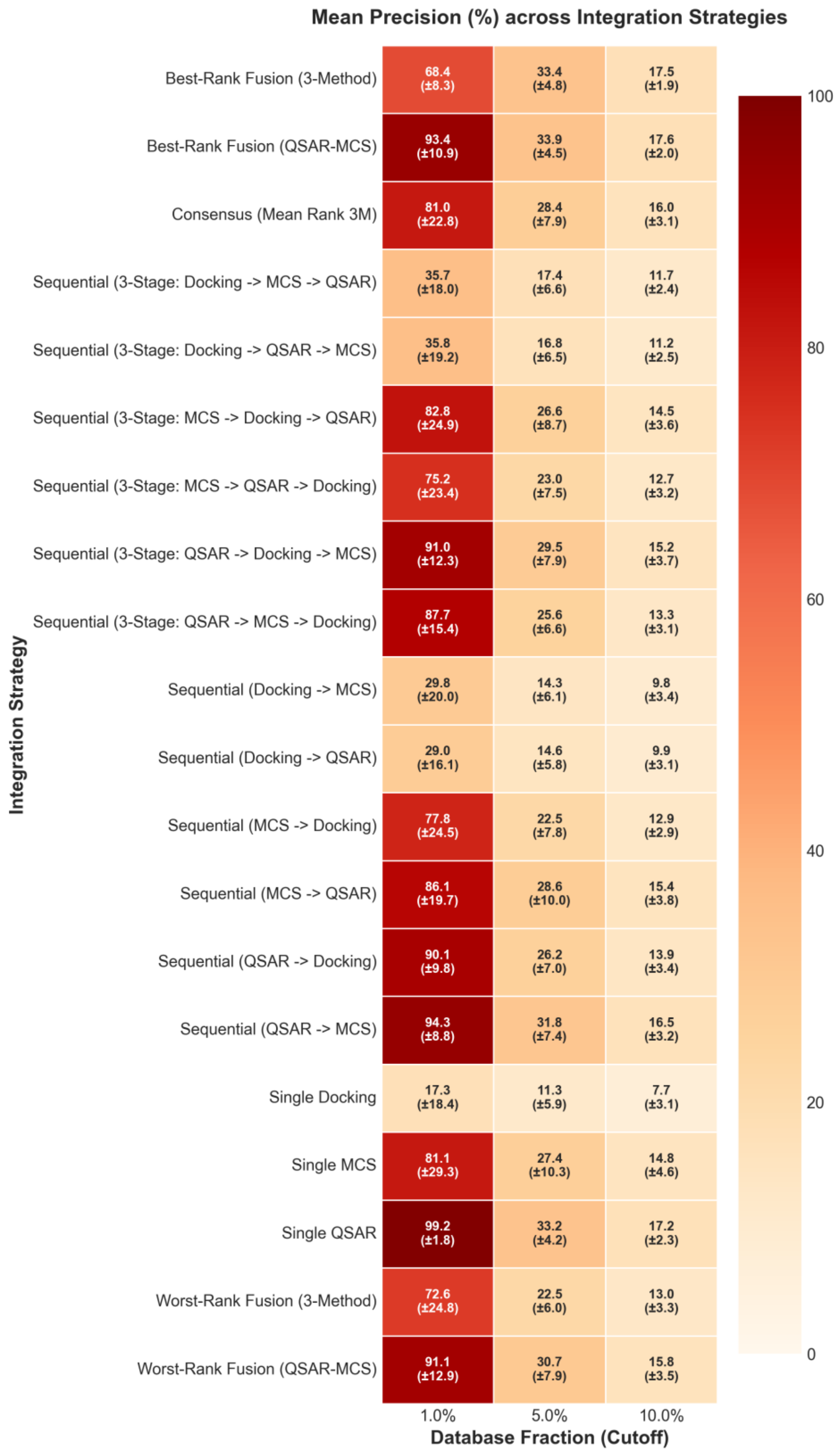

Figure S1. Comparison of VS integration strategies under a fixed experimental budget. Mean precision (%) at screening cutoffs of 1%, 5%, and 10% across all benchmark targets. Each integration strategy was constrained to select exactly the same number of compounds at every database fraction. Warmer colors indicate higher performance.

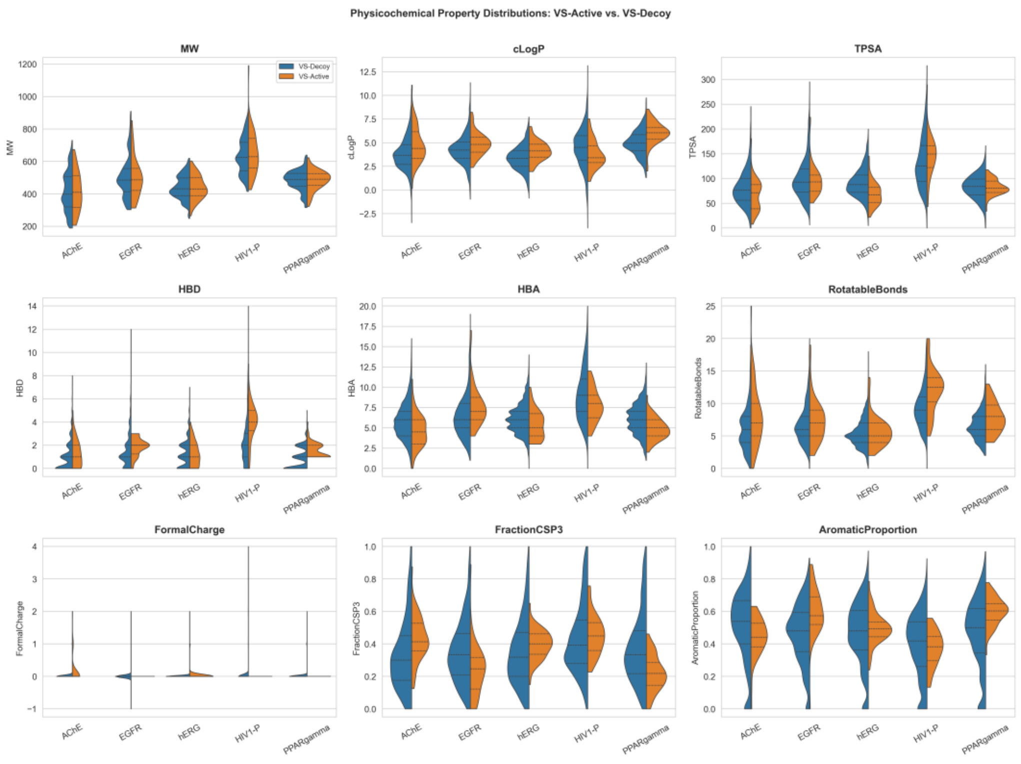

Figure S2. Comparison of physicochemical property distributions between virtual screening actives and LUDe-generated decoys. Split violin plots illustrating the distributions of nine key physicochemical properties, Molecular Weight (MW), calculated LogP (cLogP), Topological Polar Surface Area (TPSA), Hydrogen Bond Donors (HBD), Hydrogen Bond Acceptors (HBA), Rotatable Bonds, Formal Charge, Fraction of sp^3 Carbons (FractionCSP3), and Aromatic Proportion, across the five benchmark targets (AChE, EGFR, hERG, HIV1-P, and PPARγ). For each target, the right side (orange) represents the 50 selected active compounds, while the left side (blue) represents the corresponding LUDe-generated decoy set. Horizontal dashed lines inside the violins indicate the median and interquartile ranges.

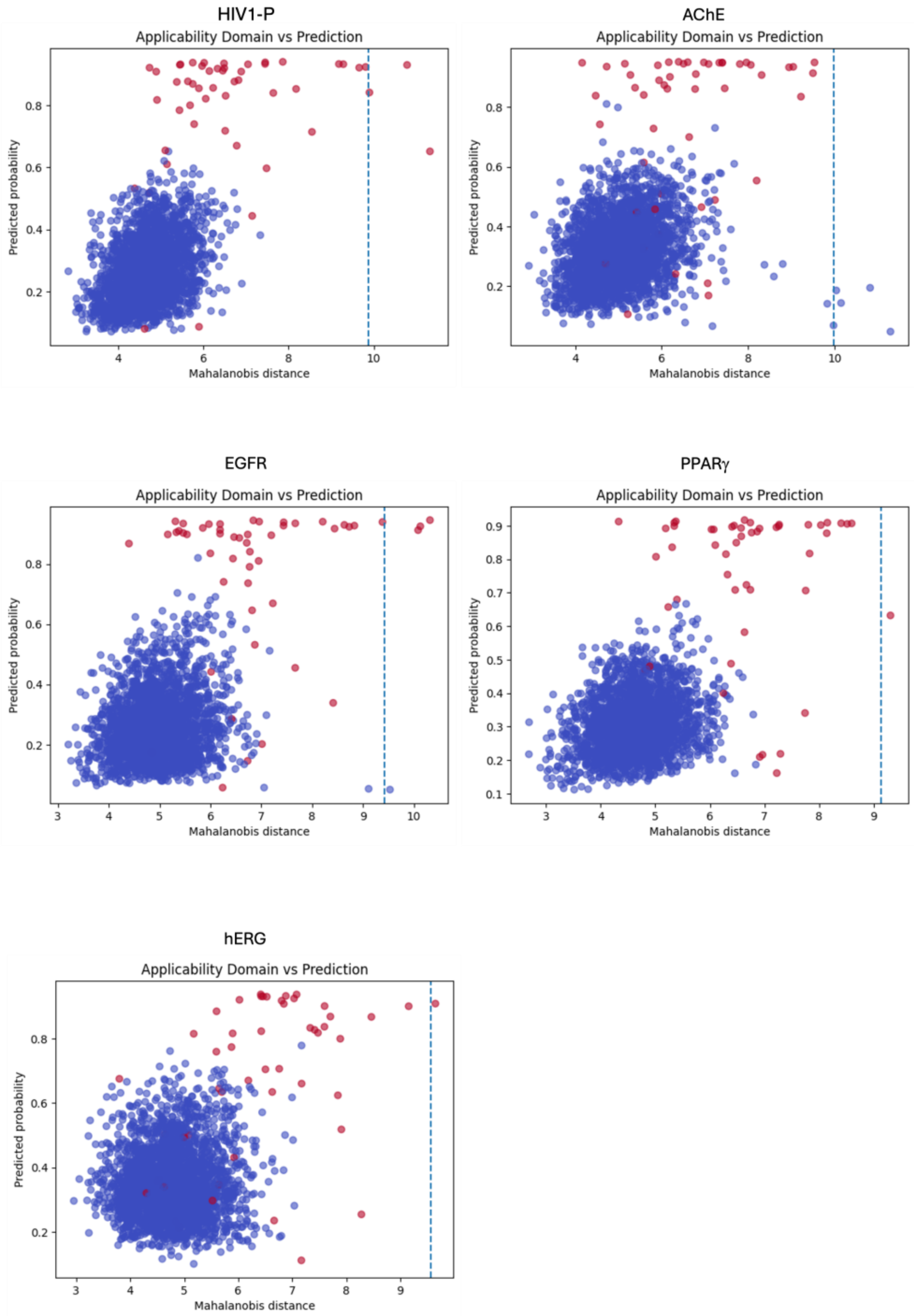

Figure S3. Applicability Domain (AD) analysis of all targets. Predicted probability plotted against the Mahalanobis distance. Red circles highlight active molecules in the virtual screening (VS) dataset, blue circles correspond to training data points, and vertical dashed lines mark the AD distance cut-off for each target.
